# Effect of Coffee Cascara Dietary Fiber on the Physicochemical, Nutritional and Sensory Properties of a Gluten-Free Bread Formulation

**DOI:** 10.3390/molecules25061358

**Published:** 2020-03-17

**Authors:** Maria Belen Rios, Amaia Iriondo-DeHond, Maite Iriondo-DeHond, Teresa Herrera, Diego Velasco, Sergio Gómez-Alonso, María Jesús Callejo, Maria Dolores del Castillo

**Affiliations:** 1 Instituto de Investigación en Ciencias de la Alimentación (CIAL) (CSIC-UAM), Calle Nicolás Cabrera, 9, 28049 Madrid, Spain; belen.rios@csic.es (M.B.R.); amaia.iriondo@csic.es (A.I.-D.); teresa.herrera@madrid.org (T.H.); 2 Instituto Madrileño de Investigación y Desarrollo Rural, Agrario y Alimentario (IMIDRA), N-II km 38, 28800 Alcalá de Henares, Spain; maite.iriondo@madrid.org; 3 Departamento de Química y Tecnología de Alimentos, Escuela Técnica Superior de Ingeniería Agronómica, Alimentaria y de Biosistemas (ETSIAAB), Universidad Politécnica de Madrid (UPM), Avenida Puerta de Hierro, nº 2, 4, 28040 Madrid, Spain; diego.velasco.arias@alumnos.upm.es (D.V.); mj.callejo@upm.es (M.J.C.); 4 Universidad de Castilla-La Mancha, Instituto Regional de Investigación Científica Aplicada (IRICA), Av. Camilo José Cela s/n, 13071 Ciudad Real, Spain; sergio.gomez@uclm.es

**Keywords:** coffee cascara, coffee cascara dietary fiber, gluten-free bread, nutrition claims, physicochemical properties, sensorial quality

## Abstract

This study aimed to assess the physicochemical, nutritional and sensory properties of gluten-free breads containing isolated coffee cascara dietary fiber (ICCDF) as a food ingredient. ICCDF was obtained by aqueous extraction. The oil and water holding capacity and the nutritional profile of the novel ingredient were determined. Its safety was certificated by analysis of ochratoxin A, caffeine and gluten. Gluten-free bread formulations were prepared enriching a commercial bakery premix in rice protein (8%) and ICCDF (3% and 4.5%). Nutritional profile of the novel gluten-free breads (dietary fiber, protein, amino acids, lipids, fatty acid profile and resistant starch), as well as bread volume, crumb density, moisture, firmness, elasticity and color intensity were determined. A sensory quantitative descriptive analysis of the breads was conducted using eight trained panelists. New breads showed significantly higher (*p* < 0.05) content of dietary fiber and protein than the control bread. The addition of ICCDF allowed increasing dough yield, a less crumb firmness and a higher crumb elasticity. The nutrition claims “source of protein and high in dietary fiber” were assigned to the new formulations. In conclusion, a certificated gluten-free bread with improved nutritional and physicochemical properties and good sensorial profile was obtained.

## 1. Introduction

According to the Food and Agriculture Organization of the United Nations, the recovery and conversion of food waste for human consumption is a main strategy for its reduction [1]. Coffee cascara (CC) is the main and first by-product generated during coffee processing. CC is generated in coffee-producing countries and constitutes a source of severe contamination causing serious environmental problems. For instance, in Colombia, one of the first producers of Arabica coffee [2], in 100 kg of mature coffee cherries, 39 kg correspond to skin and pulp [3]. 

**Figure 1:**
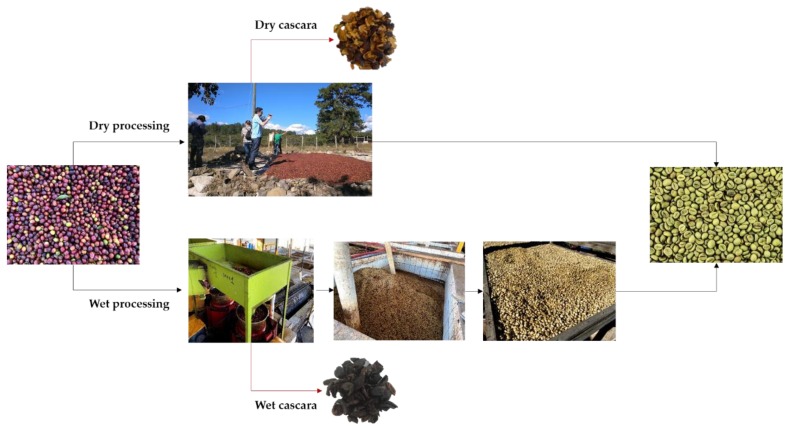
Dry (top) and wet (bottom) processing of coffee cherries. Photographs were taken in Combrifol and Café Orgánico Marcala S.A. (COMSA), Marcala, Honduras.

The composition of this by-product depends on the type of processing employed (Figure 1) [4]. CC from dry processing is composed by skin, pulp, mucilage, parchment and part of silverskin, while CC generated during the wet coffee processing consists of the skin and pulp of the coffee cherries.

Coffee cascara is currently used for composting in coffee producing countries. Other applications proposed for this by-product are production of biofuel [5], enzymes [6], biosorbents [7], particleboard [8] and animal feed [9]. In the food industry, different studies propose CC for its use as a source of anthocyanins and dietary fiber [10]. 

CC flour has been proposed as a healthy gluten-free food ingredient enriched in dietary fiber, antioxidants, iron, potassium and proteins, and has a low-fat content [11]. Pectcof B.V. has proposed the isolation of soluble dietary fiber from CC for both food and non-food applications [12]. However, CC is still considered a novel food by the European Food Safety Authority (EFSA) [13]. On the other hand, to the best of our knowledge, the isolated coffee cascara dietary fiber (ICCDF) is not commercially available. Consequently, gluten free foodstuffs such as bread containing isolated coffee cascara dietary fiber (ICCDF) as a food ingredient are not available in the European market.

Worldwide, 1% of the population is considered to suffer from celiac disease, but it is believed that the real percentage is higher [14]. Previous studies suggest that people needing a gluten free diet have an unbalanced intake of proteins, carbohydrates and fats, as well as an insufficient consumption of dietary fiber [15,16] due to the poor nutritional quality of gluten free products [17]. Therefore, gluten-free products represent a major challenge for the food industry in terms of organoleptic, technological and nutritional characteristics. The absence of gluten has been shown to affect starch digestibility, increasing the postprandial glycemic response [18]. Frequent consumption of these particular products as a therapeutic diet for these patients leads to a higher risk of developing non-communicable chronic diseases, such as diabetes [19]. Consequently, better industrial practices to achieve a low glycemic index in gluten-free products, maintaining a high sensory quality, are needed. Their nutritional quality remains a major concern. This challenge can be achieved by replacing digestible carbohydrates with dietary fiber, proteins, or using gluten-free whole grain flours [18,20].

On the other hand, the technological and sensorial properties of gluten free foodstuffs are frequently significantly different from those of traditional foods made of wheat or other gluten containing flours. Therefore, many gluten free products do not satisfy the demands of consumers needing these particular foods. The addition of dietary fiber can improve their texture and sensory characteristics, as well as prolong their shelf life due to its water-biding capacity, gel-forming potential, fat mimetic properties, and thickening effects [21,22]. Some innovative strategies include the use a wide range of hydrocolloids, enzymes, emulsifiers, proteins and fibers [23,24]. The use of ICCDF stands as a novel opportunity for the development of gluten-free products with a high nutritional value and enhanced sensory profile. Therefore, its use is of great interest for the coffee production industry as the recovery and validation of ICCDF as a value-added ingredient may contribute to increase the sustainability and product diversification of the coffee sector. 

To satisfy the demands of the consumers of healthier bread formulations based on whole grain flour, the present work aimed to assess the feasibility of the use of ICCDF and rice proteins as food ingredients for the formulation of a gluten-free bread with the nutrition claims “source of protein and high dietary fiber.” To achieve this goal, the gluten-free bread formulations were prepared according to the Global Nutrition and Policy Consortium Global Dietary Database (GDD) [25] and the new Spanish Bread Law (Royal Decree 308/2019) [26]. GDD defines a whole grain food as a food with ≥1.0 g of fiber per 10 g of carbohydrates, while the Spanish Law defines “100% whole wheat bread” or “whole wheat bread” as breads made exclusively from whole wheat flour. Rice protein is a hypoallergenic ingredient with an excellent amino acid profile and digestibility [27]. It can easily be incorporated into gluten-free baked goods to obtain high-protein products with excellent taste and texture. This is the first time that ICCDF in combination with rice protein is proposed for achieving the major food industry challenge of obtaining gluten-free products with enhanced organoleptic, technological and nutritional characteristics. Three bread formulations were prepared including 8% of rice protein (control bread B1) together with 3% and 4.5% of ICCDF flour (breads B2 and B3, respectively). The impact of the addition of the rice protein and ICCDF was evaluated on the physicochemical, nutritional and sensorial quality of the ready to eat developed foods.

## 2. Results and Discussion

### 2.1. Food Safety of Isolated Coffee Cascara Dietary Fibre and Breads

A content of 4.3 µg/kg of ochratoxin A (OTA) was detected in ICCDF. This amount is lower than the maximum level of 5 µg/kg established by the European Commission [28] in roasted coffee beans and ground roasted coffee with the exception of soluble coffee. OTA is a mycotoxin produced by *Aspergillus ochraceus* and *Penicillium verrucosum* that tends to bioaccumulate along the food chain. Coffee may be contaminated with ochratoxin A when coffee fruits fall onto the soil or during storage [29]. Values of moisture content of the samples were of 11% and 8% for CC and ICCDF, respectively. These values are lower than 13%, which is the moisture needed in stored foods for fungal growth [30]. Data are in line with those previously reported by our research group [31], supporting the biological safety of the isolated coffee cascara dietary fiber for its application as food an ingredient for human consumption. In this study, the food safety of ICCDF is also confirmed by the absence of other mycotoxins (aflatoxin B1 and enniantin B) and pesticides in this fraction [31].

Further information on safety of ICCDF was obtained by the analysis of caffeine. The content of this compound in the studied sample was of 3.2 mg/g. As expected, values were significantly lower (*p* < 0.05) than those detected in CC (7.2 mg/g) used for its extraction. This value is in the range (5.4–18 mg/g) described by other authors for this coffee by-product [32]. The EFSA considers as safe daily levels of caffeine consumption of 400 and 200 mg for the general population and in pregnant and lactating woman, respectively [33]. Therefore, the daily intake of 62.5 g of ICCDF provides a safe caffeine dose for the general public (200 mg) [34]. The World Health Organization (WHO) recommends an intake of 220–250 g of bread/person/day [35]. The intake of 250 g of bread with 4.5% of ICCDF provides approximately 36 mg of caffeine. The value is 5.5-fold lower than the level of caffeine considered as safe for pregnant and lactating woman. Therefore, caffeine levels due to the intake of breads formulated with this ingredient are safe for the general public. 

Levels of gluten lower than 3 ppm were found in breads formulated with mixtures of ingredients added or not to ICCDF. The limit set by the European Commission Regulation (EU) No. 828/2014 for the labeling of foods as “gluten-free” is 20 mg gluten/kg (ppm). Therefore, the products developed in this study can be certificated as safe for the population with the particular nutritional requirement of needing a diet excluding gluten.

### 2.2. Nutritional Analysis of CC, ICCDF and Gluten-Free Breads

#### 2.2.1. Dietary Fiber and Starch Content.

Table 1 shows values of different types of starch content and dietary fiber in CC, ICCDF and the developed gluten-free breads. No resistant starch (NRS) was detected in CC (2.19 g/100 g). The value was of the same order of magnitude than that reported in tomato peel [36]. To the best of our knowledge, this is the first time that such compound is described in CC. NRS was absent in ICCDF suggesting that it was discarded during the aqueous extraction process. 

Total starch (TS) content in the novel gluten-free breads containing ICCDF (B2 and B3) was lower than in B1. NRS content in B1 was significantly higher (*p* < 0.05) than in the gluten-free bread B3. These values may be due to the replacement of a part of the gluten-free commercial premix by ICCDF, which presented low starch content (Table 1). Regarding resistant starch (RS), no significant differences (*p* > 0.05) were found between the elaborated breads. Values of RS were lower than those obtained for gluten-free breads containing yellow pea flour, but similar to some wheat breads [37]. More studies that provide information on RS in gluten-free breads are necessary. 

A significantly higher (*p* < 0.05) value of TDF was observed in ICCDF compared to that of CC. Results suggest that dietary fiber is the major component of ICCDF obtained under the conditions hereby employed. Most of the dietary fiber retained in ICCDF was insoluble. Data on dietary fiber in CC (47.44%) and ICCDF (61.25%) are in accordance with previous published studies by The Coffee Cherry Co and others [31,38].

TDF content of the novel gluten-free breads containing ICCDF (B2 and B3) was significantly higher (*p* < 0.05) than in the control bread (B1). Significant differences (*p* < 0.05) in TDF were detected between B2 and B3. The new gluten-free breads could be labelled with the nutrition claim “high fiber content,” as they contain at least 6 g of fiber per 100 g [39]. TDF values in B2 and B3 were higher than the average gluten-free pumpernickel-style breads (5.30%) [40] and other commercial gluten-free breads (5.77%) [41]. However, still a substantial number of studies have not provided any information on fiber content in gluten-free breads [42].

Novel breads formulated in the present study contained SDF and IDF. This mixture of dietary fibers is important since SDF slows down glucose absorption, reduces plasma cholesterol concentrations, and is useful in the management of diabetes and heart disease [43,44,45]. IDF has been described to increase fecal weight, bulk, and softness, as well as the frequency of defecation, and to reduce intestinal transit times [46,47]. Therefore, the incorporation of these newly developed breads in the diet stands as a feasible a strategy to increase the intake of dietary fiber in the celiac population.

#### 2.2.2. Total Proteins and Amino Acid Profile

The amount of total proteins in CC (9.55 g/100 g) was within the expected range described by other authors (8–11 g/100 g) [5] (Table 2). Total content of proteins in ICCDF (10.96 g/100 g) was significantly higher (*p* < 0.05) than that found in CC. Regarding the newly developed breads, breads containing ICCDF (B2 and B3) presented protein values significantly higher (*p* < 0.05) compared to B1. In addition, breads enriched with ICCDF could be labelled as “source of protein” since they contained at least 12% of the energy value of the food provided by protein [39]. This may be explained by the addition of ICCDF to the novel formulations that decreased caloric content of breads (Table 5). Also, these results show higher protein values than the average protein content of gluten-free breads found in other recent studies [48].

To our knowledge, this is the first time the amino acids profile is analyzed in ICCDF. Results of essential amino acid (EAAs) in CC (33.69%) and ICCDF (33.51%) did not show significant differences (*p* > 0.05) between them. However, proteins from ICCDF contained higher levels of the EAAs isoleucine, leucine, lysine, phenylalanine, valine and histidine compared to CC. Data on amino acid content in CC are limited. Previous studies indicate that glutamic acid and aspartic acid were found mostly in CC, and cysteine and methionine presented minor values [49,50]. These values are in accordance to results obtained in this study. However, values of lysine were higher compared to data obtained in the present research (Table 2).

On the other hand, values of EAAs found in B1 (bread without ICCDF) were significantly higher (*p* < 0.05) compared to B2. In contrast, B3 did not show significant differences (*p* > 0.05) compared to B1. Results seem to indicate that the replacement of the baking pre-mix by ICCDF in B2 and B3 is insufficient to improve EAAs content. The content of aromatic amino acids (AAA) was not significantly different (*p* > 0.05) between B1, B2 and B3 breads. Most of amino acids were in higher amounts in B2 and B3, for instance arginine, which is essential for infants and children [51]. Methionine content was the lowest, while glutamic acid was the highest in B2 and B3. These results are in accordance with other recent studies on gluten-free bread made with teff [52].

#### 2.2.3. Total Fat and Fatty Acid Profile

The total fat content present in CC (2 g/100 g) was within the range (1.5–3 g/100 g) of that previously described for coffee husk [53]. Results obtained in ICCDF (2.71%) were similar to data shown by other studies [31]. However, no significant differences were found between CC and ICCDF (Table 3).

Regarding the fatty acid profile, a total of 20 and 21 fatty acids were detected in CC and ICCDF, respectively. Palmitic, linoleic (n6) and linolenic (n3) acid were the most abundant fatty acids. No trans-fatty acids were detected in the samples. The n6/n3 ratio for CC and ICCDF was 1.25 and 1.41, respectively. PUFA/SFA was higher than 0.45, as recommended by WHO [54].

The total lipid content of B1, B2 and B3 ranged between 2.51–2.95 g/100 g. No significant differences were found between breads. A total of 14 fatty acid were detected in gluten-free breads. Breads contained high values of monounsaturated (MUFA, 26–29%) and polyunsaturated fatty acids (PUFA, 47–50%). PUFA/SFA ratio was between 2.1–2.4 for B1, B2 and B3 breads. PUFA and MUFA were higher than SFA. In contrast, MUFA and PUFA content in wheat breads and gluten-free breads made with oat dietary fiber were lower than SFA [55]. Our results suggest that the newly developed breads stand as a healthy option in terms of fatty acid consumption.

### 2.3. Physical Properties of Breads

#### Water Holding Capacity (WHC) and Oil Holding Capacity (OHC)

ICCDF had significantly higher (*p* < 0.05) values of WHC (6.50%) and OHC (1.72%) than CC (2.65% and 1.01%, respectively). High WHC is associated to more elevated amounts of TDF [56]. This is in concordance with the results obtained in the present research, which showed that ICCDF presented higher TDF than CC. WHC and OHC are key properties in food processing. These properties can be defined as the capacity that a material has to retain water or oil after application of external centrifugal gravity force or compression [57]. Incorporating dietary fiber with high WHC in the diet could increase stool weight and may promote intestinal transit [58]. On the other hand, OHC plays an important role during preparation, processing and storage of foods [59].

### 2.4. Instrumental Analyses

Substituting 3% or 4.5% of gluten-free pre-mix with ICCDF did not modify bread volume or crumb density (*p* > 0.05). However, a significant decrease (*p* < 0.05) in the firmness of the crumb was observed when comparing breads B2 and B3 in relation to B1. Also, B3 showed a significant increase (*p* < 0.05) in crumb elasticity. This suggests that the addition of ICCDF could give a “softer” and more elastic bread with a longer shelf life when adding 4.5% of ICCDF compared to the control bread (B1) [60] (Table 4).

Previous studies described that the addition of dietary fiber in baked goods enhances their water absorption [61]. This explains the choice of the increased percentage of hydration in the novel formulations with ICCDF (Table 5), which allowed increasing the dough yield in these formulations. 

**Figure 2:**
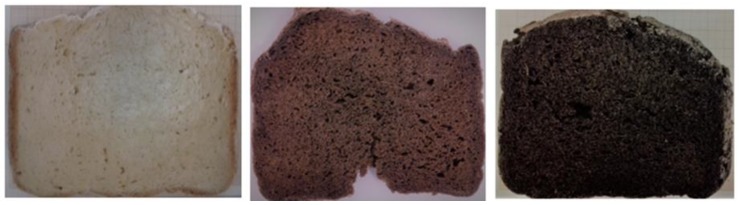
Cross section of elaborated gluten-free breads: (left) control bread (B1), (center) bread enriched with 3% of isolated coffee cascara dietary fiber (B2), (right) bread enriched with 4.5% of isolated coffee cascara dietary fiber (B3).

Crumb color was significantly darker (*p* < 0.05) in B2 and B3 breads compared to B1. The incorporation of ICCDF masked the white color of the gluten-free premix. Parameters a* and b* were significantly higher (*p* < 0.05) in B2 and B3 than in B1. The parameter L* was significantly higher (*p* < 0.05) in B1 in relation to B2 and B3. Figure 2 shows the elaborated gluten-free breads. 

Regarding to instrumental analysis, the addition of ICCDF to gluten-free breads showed a significant decrease in the crumb firmness and a significant increase in crumb elasticity. Therefore, breads with less crumb firmness and greater elasticity were obtained. On the other hand, incorporating ICCDF to gluten-free bread formulations, allowed to increase the hydration from flour and the dough yield.

### 2.5. Sensory Analysis of Breads

**Figure 3:**
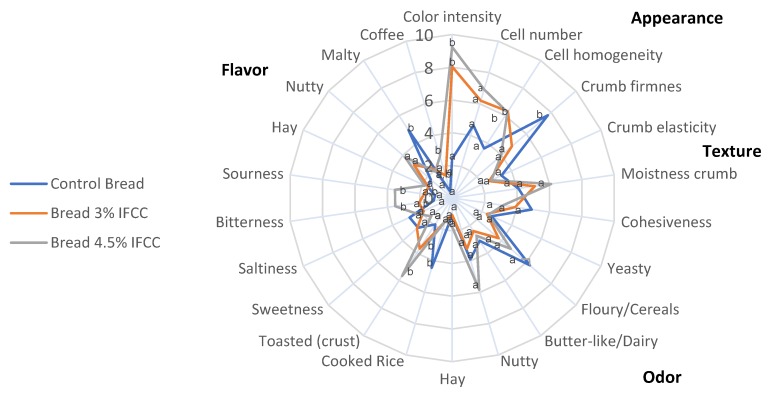
Results of the lineal scale (0–10) test of gluten-free breads by 8 trained panelists. Different letters indicate significant differences among samples (Tukey Test, *p* < 0.05).

The sensory profile of the developed breads is presented in Figure 3. In the visual phase, “color intensity” and “crumb cell homogeneity” were significantly lower (*p* < 0.05) in B1 compared to B2 and B3 breads.

In the tactile phase, “crumb firmness” was significantly lower (*p* < 0.05) in the two breads containing ICCDF (B2 and B3) compared to B1. These results are in agreement with the results obtained in the instrumental analyses. No significant differences were found (*p* > 0.05) between B2 and B3. In the olfactory phase, the addition of ICCDF produced a decrease in the attribute “cooked rice” in relation to B1, for both tested concentrations (3% and 4.5%). Significant differences were not found (*p* > 0.05) between B2 and B3. In the oral phase, the addition of 3% ICCDF did not produce a significant increase (*p* > 0.05) in “toasted (crust)”, “bitterness”, “coffee” and “sourness” attributes. However, these attributes were significantly higher (*p* < 0.05) in the bread with 4.5% (B3) compared to B1. 

The flavor “cereal” significantly decreased (*p* < 0.05) in breads with ICCDF when compared to the control bread (B1). This attribute was masked in breads with ICCDF contents by the “toasted (crust)”, “bitterness”, “sourness” attributes. Panelists detected additional attributes such as raisins, ripe figs, berries in breads made with IFCC, in the “others” section in the olfactory phase. 

Altogether, data obtained from the instrumental and sensory analyses seem to indicate that the new formulated bread would be more attractive to celiac consumers. Most of gluten-free breads are considered bland and lacking in flavor [62]. According to the Whole Grains Council, consumers consider whole-meal breads healthier [63]. Therefore, the increased color intensity associated to this type of bread would be welcomed.

## 3. Conclusions

The present research provided data for the validation of ICCDF enriched in dietary fiber (~61%) and protein (~11%), as a novel healthy food ingredient for the elaboration of gluten-free breads. The ingredient is obtained by a sustainable and low-cost process, which could be easily performed by the coffee industry. 

Novel gluten-free breads were obtained labelled as “high in dietary fiber” and “source of protein”. The two bread formulations can be defined as a whole grain food according to the GDD. In addition, the bread with 4.5% of ICCDF would correspond to a whole grain bread formulated with whole wheat flour in terms of dietary fiber concentration (~11%) without gluten and containing a mixture of soluble and insoluble dietary fiber. ICCDF had positive effects on sensory quality, due to additional attributes detected by a trained panel. Breads containing ICCDF could give a novel gluten-free bread that satisfies the consumers’ demands with improved quality and dough yield and a longer shelf life compared to the control bread. The incorporation of this novel ingredient and rice protein to a basic commercial gluten-free premix to elaborate homemade breads may be commercialized and very welcomed by the increasing celiac and gluten sensitive population. Ready-to-eat breads based on this formulation may be also commercialized but further shelf-life studies are needed. 

## 4. Materials and Methods

### 4.1. Samples

#### 4.1.1. Isolated Coffee Cascara Dietary Fiber (ICCDF)

Arabica CC, Tabi variety, was obtained by wet processing, which was provided by Supracafé S.A (Spain) origin Colombia and stored at room temperature. The ICCDF was obtained following the procedure described in the patent by del Castillo and collaborators [64]. Briefly, 75 g of CC were treated with 1.5 L boiling water for 10 min. The solid residue from the extraction process was recovered by filtration using 250 µm filters and dried in a SBANC Tray Drier (40 °C) until constant weight. Yield of ICCDF was 80%. Temperature was recorded every second in a data logger (Model HD–2178–2, Delta OHM Srl, Selvazzano Dentro, Italy) throughout the process. Then, ICCDF was milled by Variable Speed Rotor Mill Pulverisette 14 (Fritsch GmbH, Idar-Oberstein, Germany) to obtain flour.

#### 4.1.2. Breads

Three bread formulations were prepared—a control gluten-free bread (B1) and two novel formulations including ICCDF flour (B2 and B3) as described in Table 5. Breads (1 kg) were formulated using a commercial gluten-free baking basic mix (Proceli, Barcelona, Spain), rice protein (ORAFTI® Remypro N80+, Oreye, Belgium), yeast (Anchor Instant Yeast, LALLEMAND Inc., Quebec, Canada), sunflower oil (Alcampo, Madrid, Spain) and water. ICCDF flour was included as a source of dietary fiber in the novel gluten-free breads. The amount of this flour added to breads was 3% and 4.5% in order to achieve the nutritional claim “high fiber” (6 g fiber/100 g bread) [39].

Breads were baked in a domestic bread-maker (Auto Bakery FUNAI, Osaka, Japan) following the manufacturer’s instructions. Each type of bread was prepared in triplicate (*n* = 9). Solid ingredients (22 °C) were added and homogenized first, followed by the addition of liquid ingredients (10 °C) to reduce process variability during baking. Breads were stored at −20 °C until nutritional, sensory and instrumental analyses.

### 4.2. Food Safety of ICCDF

#### 4.2.1. Analysis of Mycotoxins 

Prior to the quantification of ochratoxin A (OTA) in ICCDF, the sample was ground with an A-10 Basic laboratory mill (IKA, Staufen, Germany) and stored at −20 °C until use. Ground sample (1 g) where mixed with acetonitrile:water (80:20, *v*/*v*, 2.5 mL) in a 15 mL centrifuge tube, vortexed (5 min), sonicated (3 min), vortexed again (1 min) and then samples were centrifuged (3000× *g*, 10 min). Subsequently, the supernatant was separated. The residue was extracted again following the same procedure and the supernatants were combined. One mL of the supernatant was transferred to a new 15 mL centrifuge tube and diluted with water (7 mL) to a total volume of 8 mL and centrifuged (3000× *g*, 10 min).

OTA was extracted using ISOLUTE® Myco 60 mg/3 mL (Biotage, Uppsala, Sweden) SPE columns following the procedure recommended by the supplier. Briefly, SPE column was conditioned with 2 mL acetonitrile, equilibrated with 2 mL of ammonium acetate (10 mM) and 3 mL of the diluted sample extract were loaded. Then, the column was washed with 10 mM ammonium acetate (3 mL) and with 10 mM ammonium acetate:acetonitrile (90:10, *v*/*v*, 3 mL) and dried for 30 seconds at maximum vacuum. Finally, the sample was eluted with 0.1% formic acid in acetonitrile (2 mL) and then with 0.1% formic acid in methanol (2 mL). The combined eluates were dried under vacuum (5 mbar at 35 °C) and reconstituted in 250 μL of 0.1% acetic acid in 20% acetonitrile:methanol (1:1, *v*/*v*).

HPLC-QToF analysis—the analytical system used consisted of a 1260 Infinity HPLC system coupled to a 6545 quadrupole-time of flight (Q-ToF) mass spectrometer detector (Agilent, Waldbronn, Germany). Control software was Mass Hunter Workstation (version B.06.11, Agilent Technologies, Inc., Santa Clara, CA, USA). The Q-ToF used a Dual Jet Stream Electrospray Ionization (Dual AJS-ESI) source operated in the positive ionization mode, and the following parameters were set—capillary voltage, 4000 V; fragmentor, 120 V; nozzle voltage 500 V; gas temperature, 130 °C; drying gas,13 L/min; nebulizer, 30 psig; sheath gas temperature, 300 °C; sheath gas flow, 11 L/min; acquisition range, 80–1000 *m/z*. Samples were analyzed after injection (30 μL) on a Zorbax Eclipse Plus C18 Rapid Resolution HD column (2.1 × 50 mm, 1.8 μm, Agilent, Santa Clara, CA, USA) protected with a 5 mm guard column of the same material thermostated at 30 °C. The solvents system were 5 mM ammonium formate + 0.1% formic acid in Milli-Q water (solvent A) and 5 mM ammonium formate + 0.1% formic acid in methanol (solvent B). The elution gradient was (time, % of solvent A)—0 min, 90%; 0.5 min, 90%; 10 min, 30%; 15 min, 2%; 18 min, 2%; 20 min, 90% and a post time of 5 min. Compounds were identified and quantified using the algorithm “Find by Formula” that evaluated the mass accuracy together with the isotopic relative abundance and isotopic separation.

#### 4.2.2. Caffeine

The content of caffeine was determined using capillary zone electrophoresis (CZE), as described by del Castillo and collaborators to quantify the amount of caffeine present in samples [65]. Determinations were carried out by an Agilent G1600 A (Santa Clara, CA, USA) capillary electrophoresis instrument equipped with the ChemStation software (Agilent Technologies, Inc., 2004, Santa Clara, CA, USA) and a diode array detector (DAD). The capillary was 48.5 cm long (40 cm to the detector) with an internal diameter of 50 µm and a ×3 bubble cell. Other conditions of analysis were as follows—20 mM borate buffer at pH 9.3; a voltage of 20 kV; temperature of analysis set at 25 °C; injection administered at 50 mbar for 5 s; and an electroosmotic flow (EOF) marker of acetone. Electrophoregrams (e-grams) were monitored at 280 nm, and spectra were collected from 190 to 600 nm. The capillary was conditioned after each sample was run by flushing it with 0.1 M NaOH, for 3 min, and with a buffer for another 3 min. Pure caffeine was used as a standard for identification and quantification. All analyses were performed in in duplicate for two samples of each bread; results are expressed as caffeine percentage (%).

#### 4.2.3. Gluten-Free Certification

Gluten detection and quantification was carried out by competitive ELISA. This analysis was carried out by the Proteomics Facility of the Centro Nacional de Biotecnología (CNB, CSIC, Madrid, Spain) using R5 antibody. Analyses were carried out in duplicate for two samples of each bread and results were expressed as mg of gluten per kg of bread (ppm).

### 4.3. Nutritional Analysis of CC, ICCDF and Breads

#### 4.3.1. Dietary Fiber

Insoluble (IDF), soluble (SDF) and total (TDF) dietary fiber were determined using the enzymatic-gravimetric assay based on the AOAC-991.43 and AACC-32.07.01 method. The Megazyme Total Dietary Fiber Kit (Megazyme, Wicklow, Ireland) was employed to carry out the analysis. Analysis was carried out in duplicate for two samples of each bread. Results were expressed as weight percentage (%).

#### 4.3.2. Starch

Resistant starch (RS), non-resistant starch (NRS), and total starch (TS) were detected by using a Resistant Starch Assay Kit (Megazyme, Wicklow, Ireland) following the procedure described by the manufacturer’s instructions. Analysis was carried out in duplicate for two samples of each bread. Results were expressed as weight percentage (%).

#### 4.3.3. Total Protein

Content of proteins was determined by Kjeldahl mineralization followed by a colorimetric analysis of nitrogen for quantification (AOAC-32.1.22, 920.87) according to that previously described [32]. NH_4_Cl was used for standard calibration curve. A conversion factor (5.8) was used to calculate protein content. Analysis was carried out in duplicate for two samples of each bread. Results were expressed as weight percentage (%).

#### 4.3.4. Total Amino Acids

Amino acid analysis was performed according to AOAC-994.12 method, which is based on acid hydrolysis of sample followed by HPLC with post column derivatization using ninhydrin. This analysis was performed according to that previously described [66]. Analysis was carried out in duplicate for two samples of each bread and results were expressed as nmol of amino acid.

#### 4.3.5. Total Fat

Total fat content was quantified by a gravimetric method according to Toschi and collaborators [67]. Petroleum ether was used as the extraction solvent. Analysis was carried out in duplicate for two samples of each bread. Results were as weight percentage (%).

#### 4.3.6. Fatty Acids

Fatty acid profile was obtained by gas chromatography (Agilent 7820A GC System equipped with Flame Ionization Detector, Agilent Technologies, Inc., Santa Clara, CA, USA) according to the ISO 12966-2:2017. Analysis was carried out in duplicate for two samples of each bread.

### 4.4. Physical Properties

#### 4.4.1. Moisture

Moisture content was determined by a gravimetric method as described in ISO 712:2009. Samples were weighed accurately (~5 g) into a capsule and they were dried until constant weight in an oven at 130 °C. Analysis was carried out in duplicate for two samples of each bread. Results were expressed as weight percentage (%).

#### 4.4.2. Water Holding Capacity and Oil Holding Capacity

Water holding capacity (WHC) and oil holding capacity (OHC) were determined according to Ballesteros et al. [57]. Briefly, the sample (1 g) was mixed with 10 mL of distilled water or corn oil (Fula, Sovena, Portugal; density = 0.92 g/mL). The mixtures were vortexed for 1 min, centrifuged at 2330× *g* for 30 min, and the volume of supernatant was determined. Analysis was carried out in duplicate for two samples of each bread. WHC was expressed as grams of water held per grams of sample, while OHC was expressed as grams of oil held per grams of sample.

### 4.5. Instrumental Analysis of Gluten-Free Breads

#### 4.5.1. Bread Volume

The volume was determined by the seed displacement method according to AACC 10-05: 2001. Analyses were performed in duplicate for two samples of each bread and results were expressed in mL.

#### 4.5.2. Crumb Density

Each bread was weighed in warm after baking and the crumb density was calculated as the ratio of the bread weight to the bread volume. Analysis was carried out in duplicate for two samples of each bread. Results were expressed as g/mL. After crumb density measurements breads were sliced in 6 pieces (20 mm slice thickness).

#### 4.5.3. Crumb Moisture

Crumb moisture was determined by gravimetric method as described in ISO 712:2009, at 130 °C until constant weight. Analysis was carried out in duplicate for two samples of each bread. Results were expressed as weight percentage (%).

#### 4.5.4. Texture Parameters

Crumb hardness and elasticity were measured using a Texture Analyzer (TVT-300XP) equipped with a 5 kg load cell a compression probe (T-Cy 36) with a speed at 0.5 mm/s and a distance prolongation of 20 mm. The force at the first major drop in force–deformation curve (Fmax) and deformation at maximum force were obtained in duplicate for two samples of each bread. Results of hardness are expressed as gram force (gf) and elasticity as percentage (%).

#### 4.5.5. Crumb Color

Color parameters of the crumb were expressed according to CIE L*a*b* scale [68]. Measurements were made using a PCE Instrument Spectrophotometer CSM colorimeter. Two independent measurements of a*(redness), b*(yellowness) and L*(lightness) parameters were carried out on different areas of the center of slice of bread of two different bread samples.

### 4.6. Sensory Analysis of Gluten-Free Breads

The sensory profile of the gluten-free breads (B1, B2 and B3) was carried out using a quantitative descriptive sensory analysis (QDA). Sensory evaluation was performed in one session with 8 trained panelists. Twenty-two sensory attributes were measured according to Callejo, MJ [69]. Results of the 100 mm lineal scale test were converted into a point scale scoring 0 (lowest)–9 (highest).

### 4.7. Statistical Analysis

Statistical analyses were expressed as the mean value ± standard deviation (SD). Analysis of variance (ANOVA) and the Tukey post-hoc tests were applied to determine differences between means. Differences were considered to be significant at *p* < 0.05.

## Figures and Tables

**Table 1:** Nutritional analysis of coffee cascara (CC), isolated dietary fiber from coffee cascara (ICCDF) and of gluten-free breads

Analysis	By-Products		Gluten-Free Breads		
	CC	ICCDF	B1	B2	B3
Starch					
TS (%)	2.26 ± 0.06	nd	25.02 ± 0.71 ^b^	20.01 ± 1.74 ^ab^	19.04 ± 0.93 ^a^
RS (%)	nd	nd	0.85 ± 0.01 ^a^	0.84 ± 0.16 ^a^	0.68 ± 0.06 ^a^
NRS (%)	2.19 ± 0.05	nd	24.17 ± 1.28 ^b^	19.17 ± 0.38 ^a^	18.36 ± 0.09 ^a^
Dietary fiber					
TDF (%)	47.44 ± 1.85 ^a^	61.25 ± 0.63 ^b^	1.11 ± 0.53 ^a^	6.12 ± 0.26 ^b^	11.36 ± 0.59 ^c^
IDF (%)	31.32 ± 1.51 ^a^	55.10 ± 0.30 ^b^	1.11 ± 0.53 ^a^	5.17 ± 0.16 ^b^	9.84 ± 0.06 ^c^
SDF (%)	16.12 ± 1.15 ^a^	6.20 ± 0.33 ^b^	0.00 ± 0.00 ^a^	0.95 ± 0.10 ^b^	1.52 ± 0.65 ^c^

Data are expressed as mean (*n* = 4) ± standard deviation. Statistical analyses were conducted separately for by-products and gluten-free breads. Values in each row for by-products and for breads having different letters indicate significant differences at *p* < 0.05 (Tuckey Test). TS, total starch; RS, resistant starch; NRS, non-resistant starch; TDF, total dietary fiber; IDF, insoluble dietary fiber; SDF, soluble dietary fiber; ND, not detected. B1, control bread; B2, bread with 3% ICCDF; B3, bread with 4.5% ICCDF.

**Table 2:** Total protein (%) and amino acid content (nmol) of coffee cascara (CC), insoluble coffee cascara dietary fiber (ICCDF) and breads enriched with ICCDF (B1, B2 and B3).

Analysis	By-Products		Breads		
	CC	ICCDF	B1	B2	B3
Total proteins (%)	9.55 ± 0.11 ^b^	10.96 ± 0.40 ^d^	7.25 ± 0.08 ^a^	10.02 ± 0.24 ^bc^	10.58 ± 0.14 ^cd^
Amino acids (nmol)					
Asparagine (Asn)	2.84 ± 0.14 ^a^	6.99 ± 3.33 ^b^	1.51 ± 0.25 ^a^	2.25 ± 0.21 ^a^	2.53 ± 0.49 ^a^
Threonine (Thr)	0.96 ± 0.03 ^a^	2.76 ± 1.73 ^b^	0.34 ± 0.09 ^a^	0.51 ± 0.05 ^a^	0.65 ± 0.14 ^a^
Serine (Ser)	1.91 ± 0.02 ^a^	5.46 ± 2.69 ^b^	0.98 ± 0.17 ^a^	1.63 ± 0.16 ^a^	1.88 ± 0.36 ^a^
Glutamic acid (Glu)	2.13 ± 0.04 ^a^	6.24 ± 3.58 ^b^	2.27 ± 0.59 ^a^	3.27 ± 0.28 ^ab^	3.67 ± 0.73 ^ab^
Proline (Pro)	1.60 ± 0.01 ^a^	4.10 ± 2.12 ^b^	0.82 ± 0.25 ^a^	1.24 ± 0.12 ^a^	1.50 ± 0.35 ^a^
Glycine (Gly)	2.71 ±0.17 ^a^	7.91 ± 3.39 ^b^	1.75 ± 0.20 ^a^	2.88 ± 0.34 ^a^	3.45 ± 0.68 ^a^
Alanine (Ala)	2.06 ± 0.11 ^a^	5.63 ± 2.52 ^b^	1.91 ± 0.32 ^a^	2.90 ± 0.32 ^a^	3.20 ± 0.65 ^a^
Cysteine (Cys)	0.18 ± 0.00 ^a^	0.33 ± 0.15 ^b^	0.22 ± 0.01 ^ab^	0.26 ± 0.02 ^ab^	0.22 ± 0.03 ^ab^
Valine (Val)	1.27 ± 0.02 ^a^	3.21 ± 1.64 ^b^	0.96 ± 0.15 ^a^	1.28 ± 0.10 ^a^	1.45 ± 0.27 ^a^
Methionine (Met)	0.25 ± 0.02 ^a^	0.62 ± 0.30 ^b^	0.17 ± 0.04 ^a^	0.20 ± 0.03 ^a^	0.18 ± 0.03 ^a^
Isoleucine (Ile)	0.63 ± 0.05 ^a^	1.91 ± 1.22 ^b^	0.40 ± 0.06 ^a^	0.49 ± 0.04 ^a^	0.60 ± 0.11 ^a^
Leucine (Leu)	1.17 ± 0.03 ^a^	3.33 ± 1.98 ^b^	2.66 ± 0.23 ^a^	1.28 ± 0.10 ^a^	1.58 ± 0.32 ^a^
Tyrosine (Tyr)	0.63 ± 0.05 ^a^	1.56 ± 0.68 ^b^	0.33 ± 0.07 ^a^	0.41 ± 0.11 ^a^	0.46 ± 0.11 ^a^
Phenylalanine (Phe)	0.98 ± 0.10 ^a^	2.41 ± 0.94 ^b^	0.98 ± 0.15 ^a^	1.28 ± 0.12 ^a^	1.44 ± 0.20 ^a^
Histidine (His)	0.52 ± 0.01 ^a^	1.67 ± 0.79 ^b^	0.28 ± 0.05 ^a^	0.39 ± 0.04 ^a^	0.58 ± 0.09 ^a^
Lysine (Lys)	0.38 ± 0.01 ^a^	1.41 ± 0.63 ^b^	0.56 ± 0.11 ^a^	0.82 ± 0.13 ^ab^	1.01 ± 0.23 ^ab^
Arginine (Arg)	0.48 ± 0.03 ^a^	1.43 ± 1.03 ^a^	0.38 ± 0.08 ^a^	0.43 ± 0.09 ^a^	0.75 ± 0.60 ^a^
EAA (% total)	33.69 ± 0.06 ^ab^	33.51 ± 0.78 ^ab^	35.27 ± 0.64 ^b^	32.24 ± 1.16 ^a^	32.66 ± 1.37 ^ab^
BCAA (Val + Leu + Ile) (%total)	14.83 ± 0.55 ^ab^	14.55 ± 1.17 ^ab^	15.78 ± 0.46 ^b^	14.19 ± 0.35 ^a^	14.44 ± 0.26 ^ab^
AAA (Phe + Tyr) (% total)	7.79 ± 0.70 ^a^	7.15 ± 0.76 ^a^	8.88 ± 1.07 ^a^	7.89 ± 1.02 ^a^	7.63 ± 0.70 ^a^

EAA, essential amino acids; BCAA, branched-chain amino acids; AAA, aromatic amino acids. Results are expressed as mean ± SD (*n* = 4). Statistical analyses were conducted separately for by-products and gluten-free breads. Values in each row for by-products and for breads having different letters indicate significant differences at *p* < 0.05 (Tuckey Test).

**Table 3:** Total lipids (%) and fatty acid content (g/100 g of FA methyl esters) of cascara (CC), insoluble coffee cascara dietary fiber (IFCC) and breads enriched with ICCDF (B1, B2 and B3).

Analysis	By-products		Breads		
	CC	ICCDF	B1	B2	B3
Total lipids (%)	2.00 ± 0.5 ^a^	2.71 ± 0.80 ^a^	2.95 ± 0.35 ^a^	2.75 ± 0.52 ^a^	2.51 ± 0.22 ^a^
Fatty acid profile (g/100 g)					
C12:0	0.10 ± 0.02 ^a^	0.08 ± 0.02 ^a^	N.D.	N.D.	N.D.
C14:0	1.18 ± 0.02 ^c^	1.11 ± 0.02 ^d^	0.27 ± 0.01 ^a^	0.31 ± 0.01 ^b^	0.33 ± 0.01 ^b^
C15:0	0.37 ± 0.01 ^b^	0.31 ± 0.00 ^a^	N.D.	N.D.	N.D.
C16:0	36.02 ± 0.32 ^e^	33.96 ± 0.01 ^d^	12.21 ± 0.07 ^a^	13.02 ± 0.04 ^b^	13.42 ± 0.14 ^c^
C16:1n7	3.04 ± 0.19 ^b^	3.46 ± 0.05 ^c^	0.37 ± 0.01 ^a^	0.47 ± 0.01 ^a^	0.43 ± 0.00 ^a^
C17:0	0.56 ± 0.01 ^b^	0.49 ± 0.01 ^a^	N.D.	N.D.	N.D.
C18:0	5.64 ± 0.22 ^a^	5.64 ± 0.02 ^a^	8.66 ± 0.35 ^d^	7.82 ± 0.04 ^c^	6.65 ± 0.02 ^b^
C18:1n7c	1.79 ± 0.05 ^b^	1.90 ± 0.01 ^c^	0.75 ± 0.03 ^a^	0.74 ± 0.02 ^a^	0.78 ± 0.01 ^a^
C18:1n9c	6.72 ± 0.37 ^a^	10.38 ± 0.05 ^b^	24.98 ± 0.11 ^c^	27.68 ± 0.01 ^d^	25.14 ± 0.09 ^c^
C18:2n6c	21.80 ± 0.34 ^a^	22.30 ± 0.09 ^a^	50.66 ± 0.29 ^d^	47.05 ± 0.07 ^b^	50.13 ± 0.10 ^c^
C18:3n3	17.37 ± 0.24 ^e^	15.76 ± 0.06 ^d^	0.29 ± 0.01 ^a^	0.80 ± 0.01 ^b^	1.09 ± 0.05 ^c^
C20:0	2.82 ± 0.07 ^d^	2.60 ± 0.01 ^c^	0.36 ± 0.01 ^a^	0.46 ± 0.01 ^b^	0.46 ± 0.01 ^b^
C20:1n9	0.09 ± 0.00 ^a^	0.09 ± 0.00 ^a^	0.16 ± 0.01 ^b^	0.16 ± 0.00 ^b^	0.16 ± 0.00 ^b^
C20:2n6	0.09 ± 0.00 ^a^	0.09 ± 0.00 ^a^	N.D.	N.D.	N.D.
C20:3n3	0.19 ± 0.04 ^a^	0.18 ± 0.00 ^a^	N.D.	N.D.	N.D.
C20:5n3	N.D.	0.09 ± 0.00 ^bc^	0.08 ± 0.01 ^b^	0.11 ± 0.01 ^d^	0.10 ± 0.01 ^cd^
C21:0/C20:3n6 *	0.10 ± 0.01 ^a^	0.10 ± 0.00 ^a^	N.D.	N.D.	N.D.
C22:0	0.64 ± 0.04 ^b^	0.59 ± 0.01 ^a^	0.70 ± 0.00 ^c^	0.74 ± 0.01 ^d^	0.78 ± 0.01 ^a^
C22:1n9	N.D.	N.D.	0.16 ± 0.01 ^a^	0.25 ± 0.00 ^b^	0.22 ± 0.02 ^b^
C22:6n3	0.76 ± 0.60 ^b^	0.20 ± 0.01 ^a^	N.D.	N.D.	N.D.
C23:0	0.16 ± 0.02 ^a^	0.17 ± 0.01 ^a^	N.D.	N.D.	N.D.
C24:0/C22:5n3 *	0.57 ± 0.00 ^c^	0.51 ± 0.01 ^b^	0.36 ± 0.01 ^a^	0.38 ± 0.00 ^a^	0.37 ± 0.01 ^a^
SFA	47.48 ± 0.10 ^d^	44.94 ± 0.01 ^c^	22.19 ± 0.41 ^ab^	22.35 ± 0.07 ^b^	21.57 ± 0.14 ^a^
MUFA	11.64 ± 0.12 ^a^	15.83 ± 0.11 ^b^	26.42 ± 0.12 ^c^	29.30 ± 0.02 ^e^	26.74 ± 0.09 ^d^
PUFA	40.21 ± 0.01 ^b^	38.62 ± 0.14 ^a^	50.74 ± 0.30 ^e^	47.17 ± 0.08 ^c^	50.22 ± 0.10 ^d^

SFA, saturated fatty acids; MUFA, monounsaturated fatty acids; PUFA, polyunsaturated fatty acids. Results are expressed as mean ± SD (*n* = 4). Statistical analyses were conducted separately for by-products and gluten-free breads. Values in each row for by-products and for breads having different letters indicate significant differences at *p* < 0.05 (Tuckey Test). N.D, Not detected. * The chromatographic method does not allow the separation of the fatty acids C21:0 and C20:3n6; and C24:0 and C22:5n3, so the value obtained may be due to either one or the sum of both.

**Table 4:** Instrumental analysis of control breads (B1) and breads enriched with 3% and 4.5% of the ICCDF (B2 and B3 respectively).

Parameters	Gluten-Free Breads		
	B1	B2	B3
Volume (mL)	1527.50 ± 208.60 ^a^	1800.00 ± 169.71 ^a^	1715.00 ± 162.63 ^a^
Crumb density (g/mL)	0.65 ± 0.10 ^a^	0.55 ± 0.05 ^a^	0.57 ± 0.06 ^a^
Crumb moisture (%)	45.50 ± 1.22 ^a^	55.35 ± 0.06 ^b^	58.54 ± 0.42 ^c^
Texture			
Hardness (gf)	5585.00 ± 726.69 ^b^	2046.63 ± 323.33 ^a^	1560.75 ± 202.87 ^a^
Elasticity (%)	16.00 ± 4.08 ^a^	13.00 ± 4.98 ^a^	26.50 ± 3.10 ^b^
Color			
L *	80.32 ± 0.98 ^c^	39.95 ± 1.72 ^b^	36.22 ± 2.86 ^a^
a *	0.19 ± 0.34 ^a^	9.49 ± 0.27 ^b^	9.46 ± 0.30 ^b^
b *	16.77 ± 0.52 ^a^	20.80 ± 0.52 ^b^	19.70 ± 0.38 ^b^

Results are expressed as mean ± standard deviation (*n* = 4). Different letters denote statistically significant differences between samples (Tuckey test, *p* < 0.05).

**Table 5:** Bread formulations of control bread (B1), bread with 3% isolated coffee cascara dietary fiber (B2) and bread with 4.5% isolated coffee cascara dietary fiber (B3).

Ingredients (g)	Gluten-Free Breads		
	B1	B2	B3
Gluten-free baking pre-mix	547	387	331
ICCDF	0	57	79
Rice protein	55	55	51
Yeast	5	5	5
Sunflower oil	19	19	18
Water	422	525	565
Estimated calories (kcal/100 g bread)	229	185	167
Estimated protein energetic value (% on total kcal)	8.6	11.7 ^‡^	12.8 ^‡^
Estimated fiber content (% fiber/100 g bread)	2.8	5.5 ^ɸ^	6.6 ^ɸ^

^‡^ B2, B3 might be “source of protein” (≥12% energy value of bread provided by protein); ^ɸ^ B2 and B3 might be “high fiber content” (≥6 g fiber/100 g bread).
